# O Tratamento Medicamentoso Habitual é Suficiente para Manter o Controle da Frequência Cardíaca nos Pacientes com Insuficiência Cardíaca?

**DOI:** 10.36660/abc.20190090

**Published:** 2020-12-01

**Authors:** Juliano Cardoso, Mateus Dressler de Espíndola, Mauricio Cunha, Enock Netto, Cristina Cardoso, Milena Novaes, Carlos Henrique Del Carlo, Euler Brancalhão, Alessandro Lyra Name, Antonio Carlos Pereira Barretto

**Affiliations:** 1 Hospital Santa Marcelina São PauloSP Brazil Hospital Santa Marcelina – Cardiologia, São Paulo, SP – Brazil

**Keywords:** Insuficiência Cardíaca, Frequência Cardíaca, Tratamento Farmacológico, Adesão à Medicação, Digoxicina, Morbidade e Mortalidade, Fibrilação Atrial, Marca-Passo

## Abstract

**Fundamento:**

Estudos revelam que pacientes com insuficiência cardíaca (IC) e frequência cardíaca (FC) <70 batimentos por minuto (bpm) evoluem melhor e têm menor morbimortalidade em comparação com FC >70. Entretanto, muitos pacientes com IC mantêm FC elevada.

**Objetivo:**

Avaliar se os pacientes acompanhados em ambulatório de cardiologia têm sua FC controlada e como estava a prescrição dos medicamentos que reduzem a mortalidade na IC.

**Métodos:**

Foram analisados de forma consecutiva pacientes que passaram em consulta e que já acompanhavam em ambulatório de cardiologia, idade > 18 anos e com diagnóstico de IC e fração de ejeção do ventrículo esquerdo (FEVE) <45%. Os pacientes em ritmo sinusal foram divididos em dois grupos: FC ≤70 bpm (G1) e FC >70 bpm (G2). Na análise estatística, foram utilizados os testes t de Student, Qui-quadrado. Foi considerado significante p <0,05. Utilizamos o programa Statistical Package for the Social Sciences (SPSS) para análise.

**Resultados:**

Foram avaliados 212 pacientes de forma consecutiva. Destes, 41 (19,3%) apresentavam fibrilação atrial ou eram portadores de marca-passo e foram excluídos desta análise; assim, 171 pacientes foram analisados. Os pacientes em ritmo sinusal tinham idade média de 63,80 anos (±11,77), sendo 59,6% homens e FEVE média de 36,64% (±7,79). Com relação à etiologia, a isquêmica estava presente em 102 pacientes (59,65%), enquanto a cardiopatia chagásica em 17 pacientes (9,9%); 131 pacientes eram hipertensos (76,6%), enquanto 63 pacientes (36,84%) eram diabéticos. Quanto à FC, 101 pacientes apresentaram FC ≤70 bpm (59,06%) G1 e 70 pacientes (40,93%) FC >70 bpm (G2). A FC média no G1 foi de 61,53 bpm (±5,26) e no G2, 81,76 bpm (±9,52), p <0,001. A quase totalidade dos pacientes (98,8%) estava sendo tratada com carvedilol prescrito na dose média de 42,14 mg/dia (±18,55) no G1 *versus* 42,48 mg/dia (±21,14) no G2, p=0,911. A digoxina foi utilizada em 5,9% dos pacientes no G1 *versus* 8,5% no G2, p=0,510. A dose média de digoxina no G1 foi de 0,19 mg/dia (±0,06) e no G2 foi de 0,19 mg/dia (±0,06), p=0,999. A maioria dos pacientes (87,72%) utilizou o inibidor da enzima de conversão da angiotensina (IECA) ou bloqueador do receptor da angiotensina (BRA), e 56,72% utilizaram espironolactona. A dose média de enalapril foi de 28,86 mg/dia (±12,68) e de BRA foi de 87,80 mg/dia (±29,80). A maioria dos pacientes utilizou IECA ou BRA e com doses adequadas.

**Conclusão:**

O estudo revelou que 40,93% dos pacientes estavam com FC acima de 70 bpm, apesar de o betabloqueador ter sido prescrito para praticamente todos os pacientes e em doses elevadas. Outras medidas precisam ser adotadas para manter a FC mais controlada nesse grupo de frequência mais elevada. (Arq Bras Cardiol. 2020; 115(6):1063-1069)

## Introdução

A insuficiência cardíaca (IC) é uma síndrome cada vez mais frequente que evolui com elevada morbidade e mortalidade nas formas avançadas, sendo a fase final comum das cardiopatias.^1^

Apesar da gravidade da doença, o tratamento correto conforme as diretrizes melhora a qualidade de vida e reduz a mortalidade.^2 , 3^ Apesar das evidências da sua efetividade, a prescrição dos medicamentos que modificam o prognóstico é inferior ao desejado, como demonstrado em alguns registros recentes.^4 - 6^ Ao analisar os potenciais motivos para a subprescrição de fármacos direcionados aos pacientes com IC, é possível destacar hipotensão, idade avançada, além do receio de possíveis efeitos colaterais.^6 , 7^ Outro provável motivo para não alcançar as doses de eficácia comprovada pode ser a falta de metas objetivas no tratamento da IC, assim como fazemos no tratamento das dislipidemias e da hipertensão arterial.^8 , 9^ Talvez devêssemos perseguir no tratamento da IC algumas metas objetivas, como o controle mais rígido da frequência cardíaca (FC). A FC nos pacientes com IC tem se mostrado um guia importante para avaliação de eficácia do tratamento. Para os pacientes com IC, fração de ejeção do ventrículo esquerdo (FEVE) reduzida e em ritmo sinusal, o estudo SHIFT documentou que reduzir a FC para valores inferiores a 70 batimentos por minuto (bpm) modifica o prognóstico. Entretanto, uma parcela dos nossos pacientes ainda apresenta FC acima do desejado.^10^

Neste trabalho, procuramos verificar em um ambulatório de um grande hospital terciário da zona leste da cidade de São Paulo se os pacientes em ritmo sinusal apresentavam FC controlada (FC ≤70 bpm). Também avaliamos se a medicação para IC estava adequada conforme as diretrizes.^2^

## Material e Métodos

Para realizar esta pesquisa avaliamos os pacientes com IC que foram atendidos consecutivamente no ambulatório de cardiologia no período de janeiro de 2016 a março de 2017 com FEVE <45%, em tratamento da IC há mais de 6 meses. Avaliamos dados demográficos, etiologia da cardiopatia, ritmo cardíaco, pressão arterial, FC e o tratamento medicamentoso, verificando as doses alcançadas com os diferentes fármacos.

Os critérios de inclusão foram: idade >18 anos, diagnóstico de IC, FEVE <45% e ritmo sinusal. Para fim de análise, os pacientes foram divididos em dois grupos, um com FC ≤70 bpm (G1) e outro com FC >70 bpm (G2).

Foi avaliada a prescrição dos três grupos de medicações que comprovadamente modificam a história natural da IC: vasodilatadores (inibidor da ECA [IECA] ou bloqueador dos receptores da angiotensina II [BRA] ou hidralazina e nitrato), espironolactona e betabloqueador; somente naqueles com disfunção renal e com hiperpotassemia persistente e que não toleravam pelo menos 50% de IECA/BRA, os vasodilatadores utilizados eram hidralazina e nitrato. Já nos pacientes em uso de dose plena de IECA/BRA, betabloqueador e espironolactona e que permaneciam sintomáticos, era adicionado ao tratamento o uso de hidralazina e nitrato.

A dose considerada correta para o IECA foi de 20 mg 2x/dia de enalapril ou dose equivalente de captopril (150 mg/dia). Para os BRA, a dose considerada correta foi de 100 a 150 mg/dia de losartana. Para espironolactona, a dose plena foi de 25 mg/dia. Para os betabloqueadores, a dose considerada plena foi de 25 mg 2x/dia para o carvedilol.^2^ Identificaram-se também o percentual e a dose de prescrição de digoxina, hidroclorotiazida e furosemida, medicações frequentemente prescritas para os pacientes com IC.^2^

Para realização deste trabalho, houve aprovação do projeto pelo Comitê de Ética e Pesquisa da Casa de Saúde Santa Marcelina, sob parecer n° 13.10.805.

### Análise Estatística

Na apresentação das características da população, as variáveis contínuas com distribuição normal são apresentadas como média ± desvio padrão. As variáveis categóricas são apresentadas como número (porcentagem). O teste de Kolmogorov-Smirnov foi utilizado para testar a normalidade dos dados (p >0,05 = distribuição normal). Na comparação dos grupos, as variáveis contínuas são apresentadas como média ± desvio padrão. Foi utilizado o teste *t* -Student não pareado para as variáveis com distribuição normal. Os testes utilizados foram bicaudais e o valor de p <0,05 foi considerado como estatisticamente significante. Na comparação das características, foram utilizados os testes do Qui-quadrado ou Teste Exato de Fisher para as variáveis categóricas. Todas as análises estatísticas foram realizadas com *o software* estatístico Statistical Package for the Social Sciences (SPSS).

## Resultados

Foram analisados 212 pacientes de forma consecutiva. Deste grupo, 41 (19,3%) apresentavam fibrilação atrial ou eram portadores de marca-passo e foram excluídos desta análise. Portanto, analisamos 171 pacientes em ritmo sinusal. A idade média foi de 63,80 anos ± 11,77, sendo 59,6% homens, FEVE média 36,64% ± 7,79 e Pro-BNP médio de 1663,95 pg/mL ± 2158,77. A etiologia isquêmica ocorreu em 102 pacientes (59,65%). Diabetes estava presente em 63 pacientes (36,84%), e 131 pacientes (76,6%) apresentavam hipertensão arterial. Na Tabela 1 , apresentamos as características clínicas dos pacientes e o tratamento que vinham recebendo. Na Tabela 2 , apresentamos as doses dos medicamentos prescritos (mg/dia).

**Tabela 1:** – Características clínicas e tratamento dos pacientes em ritmo sinusal estudados.

Pacientes n	171
Idade em anos	63,80 ± 11,77
Isquêmica	102 (59,65%)
Chagásica	17 (9,9%)
Idiopática	29 (17%)
**Comorbidades n**	
Diabetes	63 (36,84%)
Hipertensão arterial	131 (76,6%)
Dados clínicos	
PAS em mmHg	119,56 ± 18,69
FC em bpm	69,81 ±12,34
NT-proBNP	1663,95 ± 2158,77
**Dados do ecocardiograma**	
DDVE em mm	61,34 ± 7,79
DSVE em mm	50,33 ± 8,25
FEVE percentual	36,64 ± 6,73
**Medicamentos prescritos n**	
Furosemida	90 (52,63%)
Hidroclorotiazida	25 (14,61%)
IECA/BRA	150 (87,72%)
Betabloqueador	169 (98,83%)
Espironolactona	97 (56,72%)
Hidralazina	30 (17,54%)
Nitrato	42 (24,56%)
Digoxina	12 (7,01%)


*PAS: pressão arterial sistólica; FC: frequência cardíaca; DDVE: diâmetro diastólico do ventrículo esquerdo; DSVE: diâmetro sistólico do ventrículo esquerdo; FEVE: fração de ejeção do ventrículo esquerdo; NT-proBNP: porção N terminal do peptídio natriurético do tipo “B”; IECA: inibidor da enzima conversora da angiotensina; BRA: bloqueador do receptor da angiotensina.*

**Tabela 2:** – Dose média dos medicamentos prescritos (mg/dia) e desvio padrão

Medicamento	Dose
Furosemida	52,31 ± 26,15
Hidroclotiazida	26,09 ± 5,10
Enalapril	28,86 ± 12,68
Losartana	87,80 ± 29,80
Carvedilol	42,28 ± 19,65
Espironolactona	25,00 ± 4,77
Hidralazina	96,55 ± 59,35
Isossorbida	53,90 ± 15,60
Digoxina	0,19 ± 0,06

Na Tabela 3 , estão demonstradas as diferenças entre os grupos de acordo com a frequência cardíaca.

**Tabela 3:** – Dados clínicos e de tratamento dos pacientes em ritmo sinusal de acordo com FC

	G1 (FC ≤70bpm)	G2 FC >70 bpm	p
**Pacientes**	101 (59,06%)	70 (40,93%)	
Homens	62 (61,38%)	40 (57,97%)	
**Etiologia da cardiopatia**			
Isquêmica	59 (58,41%)	43 (61,64%)	0,938
Chagásica	16 (15,84%)	3 (4,28%)	0,009
Não isquêmica	26 (25,74%)	24 (34,28%)	
**Comorbidades**			
Diabetes	31 (30,69%)	32 (45,71%)	0,045
Hipertensão arterial	82 (81,18%)	50 (71,42%)	0,108
**Dados clínicos**			
PAS em mmHg	119,76 ± 17,87	119,29 ± 19,81	0,871
FC em bpm	61,53 ± 5,26	81,76 ± 9,52	<0,001
NT-proBNP	1625,09 ± 2258,42	1721,80 ± 1999,91	0,822
**Dados do Ecocardiograma**			
DDVE em mm	61,26 ± 7,78	61,46 ± 7,82	0,868
DSVE em mm	49,84 ± 8,42	51,12 ± 7,92	0,356
FEVE em %	37,46 ± 6,58	35,46 ± 6,78	0,056
**Dose dos medicamentos prescritos**			
Furosemida	50,57 ± 25,06	54,74 ± 27,41	0,458
Hidroclorotiazida	26,92 ± 6,66	25,00 ± 0,00	0,392
Enalapril	29,77 ± 12,38	27,50 ± 12,99	0,361
Losartana	80,43 ± 33,75	97,22 ± 20,22	0,076
Carvedilol	42,14 ± 18,55	42,48 ± 21,14	0,911
Espironolactona	24,79 ± 4,84	25,35 ± 4,64	0,585
Hidralazina	111,11 ± 67,81	72,73 ± 29,11	0,097
Isossorbida	55,77 ± 16,21	50,67 ± 13,89	0,325
Digoxina	0,19 ± 0,06	0,19 ± 0,06	0,999

*PAS: pressão arterial sistólica; FC: frequência cardíaca; DDVE: diâmetro diastólico do ventrículo esquerdo; DSVE: diâmetro sistólico do ventrículo esquerdo; FEVE: fração de ejeção do ventrículo esquerdo; NT-proBNP: porção N terminal do peptídeo natriurético do tipo “B”.*

## Discussão

Nosso estudo revelou que 40,93% dos pacientes avaliados apresentavam FC maior que 70 bpm, apesar de o carvedilol ter sido utilizado em mais de 98% dos pacientes e com dose média elevada, acima de 42 mg/dia. Os dados deste estudo revelam que, na maioria dos casos, foi possível prescrever corretamente os medicamentos que modificam o prognóstico na IC. A frequência de prescrição foi mais elevada que as descritas nos registros internacionais, bem como no Registro Brasileiro BREATHE.^4 - 6^ Os pacientes estavam recebendo, em sua maioria, as doses-alvo dos medicamentos, como indicado pelas diretrizes.^2 , 3^

Esses resultados permitem supor que a não prescrição dos medicamentos pelos médicos, na maioria das vezes, decorre da falta de tentativa em aumentar as doses. É importante ressaltar que a não prescrição em doses, de pelo menos 50% da dose-alvo dos medicamentos, implica menor proteção aos pacientes e um risco maior de morte e hospitalizações.^6 , 7^

**Figura 1:**
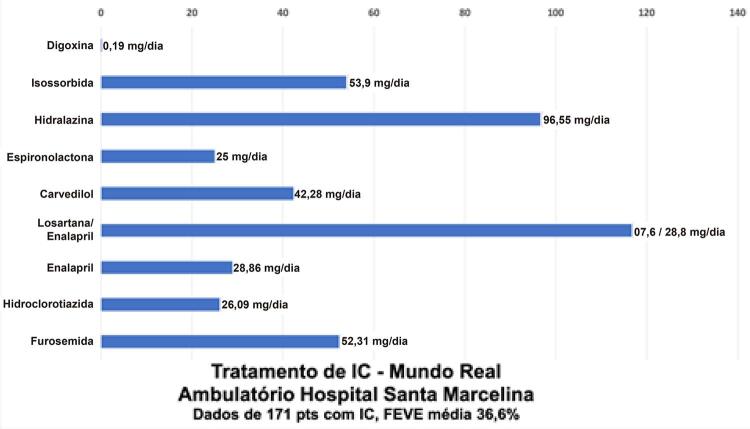
– Gráfico mostrando o percentual de pacientes tomando cada medicamento (%) e a dose média prescrita para cada fármaco.

Nossos dados diferem dos dados de Registros como o BIOSTAT-HF, no qual somente 60% dos pacientes estavam recebendo dose de 50% ou mais da dose-alvo de IECA ou BRA, e cerca de 40% recebiam dose de 50% ou mais da dose-alvo de betabloqueador, doses estas associadas à redução de mortalidade, como bem demonstrado nesse Registro.^6^ O mesmo se aplica para dados brasileiros quando o Registro BREATHE mostrou que 83,4% estavam recebendo IECA/BRA na alta do hospital e 63,1%, um betabloqueador.^5^ No Registro QUALIFY, apesar de a maioria dos pacientes estar recebendo prescrição de IECA/BRA (87,5%) ou betabloqueador (87%), somente 14,8% recebiam a dose-alvo e 51,8% recebiam dose superior a 50% da dose-alvo de IECA. Ao passo que, nesse mesmo estudo, 27,9% estavam recebendo dose-alvo de betabloqueador e 51,8% receberam dose 50% ou mais da dose-alvo do betabloqueador.^4^ Em nossos pacientes, 79,09% estão recebendo dose-alvo de IECA, sendo que 53,63% destes estavam recebendo 40 mg/dia de enalapril, e 58,47% recebiam dose-alvo de betabloqueador, sendo que 15% destes, com doses superiores a 50 mg/dia do carvedilol. Uma dose de 50% ou mais da dose-alvo de IECA foi prescrita para 97,27% dos pacientes e 88,88% recebiam dose de 50% ou mais de betabloqueador (carvedilol) ( Figura 1 ).

**Figura 2:**
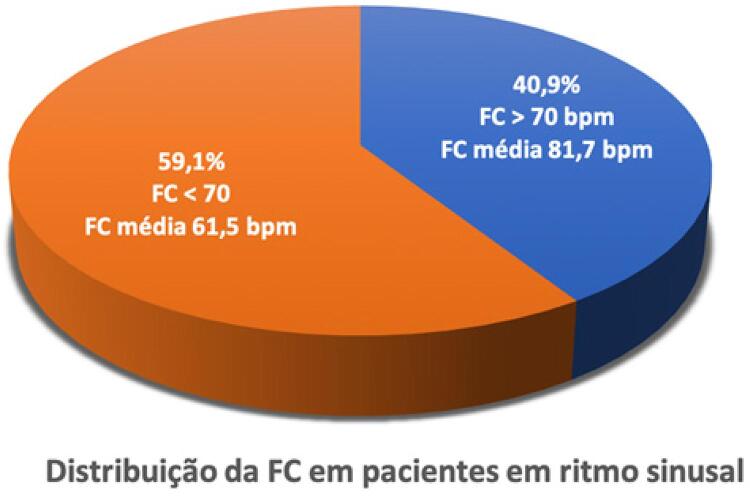
– Percentual de pacientes em ritmo sinusal que apresentam frequência cardíaca (FC) maior ou menor que 70 bpm.

Nosso estudo revelou também que mesmo bem tratados com dose média de carvedilol de 42,48 mg/dia, muitos pacientes ainda apresentam FC acima de 70 bpm; 40% em ritmo sinusal apresentavam FC >70 bpm ( Figura 2 ). Esses resultados estão de acordo com os da literatura, pois a totalidade dos artigos que aborda FC em pacientes em tratamento da IC com IECA, betabloqueador e espironolactona descreve que percentual acentuado de pacientes continua com FC acima de 70 bpm, apesar do tratamento. Vale ressaltar que, em muitos desses artigos, a dose de betabloqueador não é elevada, com a maioria permanece com dose abaixo de 50% da dose-alvo.^6 , 7^

No Registro OPTIMIZE-HF, que avaliou 10.697 pacientes de hospitais dos EUA, constatou-se que a FC média na alta era de 76 bpm, e que não houve uma correlação com a dose do betabloqueador. Os pacientes com dose inferior a 25% da dose-alvo de betabloqueador apresentaram FC média de 78 bpm, já os com a dose-alvo de 72 bpm, a FC elevada guardou relação com o prognóstico, sendo maior a morbimortalidade para aqueles com FC acima de 70 bpm.^11^ Na Duke University, verificou-se também que a maioria dos pacientes (73%) tratados apresentava FC acima de 70 bpm. Tais pacientes com FC acima de 70 bpm apresentaram maior morbimortalidade (RR 1,59), e foi observado que a FC elevada foi associada a maior custo de tratamento.^12^ Habal et al.^13^ verificou que o risco de morte dos pacientes era 59% maior se os pacientes apresentassem FC acima de 90 bpm em relação aos com FC entre 61 a 70 bpm.^13^ O estudo ASCEND-HF revelou que muitos pacientes persistiam com FC elevada, sendo que 85% dos pacientes apresentavam FC acima de 70 bpm, e esta FC elevada foi associada à maior mortalidade.^14^

No nosso estudo, embora expressivo o número de casos com FC >70 bpm, ela foi menor que a descrita nesses estudos comentados, possivelmente em decorrência do tratamento com doses mais elevadas dos betabloqueadores. Tal fato também foi observado em estudo com pacientes de consultório que estavam recebendo dose próxima à dose-alvo de carvedilol, no qual encontramos cerca de 35% dos pacientes com FC acima de 70 bpm.^15^

Um tema em discussão na literatura é o que seria mais importante para determinar uma boa evolução dos portadores de IC: se a dose-alvo do betabloqueador ou a redução da FC. Vale ressaltar que a redução da FC com os betabloqueadores não é igual para todos os pacientes. No estudo MERIT-HF, identificaram-se dois grupos: um que teve redução da FC com doses baixas de metoprolol (dose média 76 mg/dia) e outro que necessitou da dose-alvo para reduzir a FC (dose média 195 mg/dia).^16^ Essa possível diferença pode ser geneticamente determinada,^16^ sendo alguns pacientes muito responsivos às doses dos medicamentos.^16^ Quanto aos resultados do estudo, os autores sinalizaram que a redução de eventos não foi estatisticamente diferente nos dois grupos, indicando que a redução de FC foi mais importante que a dose do betabloqueador na redução de eventos cardiovasculares.^16^

Considerando-se a redução da FC, é importante ressaltar que o estudo SHIFT indicou que a FC-alvo seria inferior a 70 bpm.^10^ No próprio estudo SHIFT, também se documentou que a redução da FC foi mais importante que a dose do betabloqueador na promoção da redução de eventos cardiovasculares.^17^

Quando se aborda a FC, é importante ressaltar que a maior redução de eventos tem sido descrita quando se atinge FC inferior a 64 bpm, como bem demonstrado nos estudos CHARM e CIBIS-ELD.^18 , 19^

Em metanálise com vários ensaios clínicos com betabloqueador, a sua prescrição foi associada à redução de mortalidade de 34%, e observou-se que a redução da FC se correlacionou melhor com a redução de eventos do que com a dose do betabloqueador.^20^ Nesta metanálise para a redução de cada 5 bpm, observou-se redução de 18% no risco de morte e, quanto à dose, ela não foi determinante da redução de eventos, constatando-se redução de mortalidade de 26% para dose mais elevada e de 22% para as doses mais baixas.^20^

Nos estudos BIOSTAT-HF e na análise retrospectiva dos dados do estudo ACTION-HF, observou-se maior redução de eventos nos pacientes tratados com dose mais elevada do betabloqueador.^6 , 21^ No estudo BIOSTAT-HF, doses superiores a 50% da dose-alvo foram associadas à maior redução de mortalidade entre os 2.516 pacientes estudados. Os autores, na discussão, comentam que não conseguiram detectar diferença na evolução entre os tratados com mais de 50% da dose-alvo e aqueles tratados com dose alvo, mas doses mais baixas não protegeram os pacientes.^6^ No estudo ACTION-HF, os pacientes que tiveram a melhor evolução (maior redução de mortalidade) foram aqueles que tiveram redução da FC abaixo de 70 bpm com dose de 50% ou mais do betabloqueador. Os pacientes com dose mais baixa tiveram maior mortalidade. Quando se analisa somente os pacientes com dose mais baixa, aqueles que apresentavam FC menor que 70 bpm tiveram melhor evolução do que aqueles com FC acima de 70 bpm.^21^

Podemos concluir que os dois pontos são importantes, doses baixas de betabloqueador e FC acima de 70 bpm estão associadas a pior prognóstico. Os dados da literatura destacam a importância de avaliar a FC em todos os pacientes e, naqueles com FC acima de 70 bpm, otimizar o tratamento, quer aumentando a dose do betabloqueador se estiver sendo prescrita em dose baixa quer prescrevendo ivabradina para que seja possível reduzi-la, uma vez que a FC acima de 70 bpm vem se mostrando um excelente e fácil marcador de pior evolução. Vale lembrar que quanto mais elevada a FC, pior o prognóstico. Devemos nos policiar para evitar a inércia de deixar para a próxima avaliação a mudança de conduta quando encontramos um paciente em ritmo sinusal com FC acima de 70 bpm. É importante também lembrar que doses baixas de betabloqueador não tiveram sua eficácia comprovada. Além disso, nos pacientes que apresentam FC elevada apesar do uso da dose correta de betabloqueador, podemos utilizar a ivabradina, que é um inibidor dos canais *f* e que reduz a FC nos pacientes em ritmo sinusal.^2^

Tivemos como limitação deste estudo o fato de ser unicêntrico, tendo, portanto, as limitações relacionadas a esse fato. O estudo teve como ponto forte revelar que, dentre os pacientes estudados, apesar de serem bem tratados do ponto de vista da IC, inclusive com doses adequadas de betabloqueador, muitos ainda permanecem com FC mais elevada.^22^ Fato que relevante, pois está relacionado com o prognóstico desses pacientes

## Conclusão

Dos pacientes avaliados em ritmo sinusal, 40,93% ainda apresentavam FC acima de 70 bpm, apesar de o betabloqueador ter sido prescrito para 98,83% dos pacientes, e com dose média elevada (42,28mg/dia + 19,65). Outras medidas precisam ser adotadas para manter a FC mais controlada nesse grupo que persiste com a FC elevada. Os vasodilatadores foram utilizados com frequência e com dose média adequada.
