# Supplementary material for: O Tratamento Medicamentoso Habitual é Suficiente para Manter o Controle da Frequência Cardíaca nos Pacientes com Insuficiência Cardíaca?
Source: Arq Bras Cardiol. 2020 Dec 1;115(6):1063–9. [Article in Portuguese] doi: 10.36660/abc.20190090 (PMC8133726; doi:10.36660/abc.20190090)
Supplement: Translation

# Translation: Is Current Drug Therapy for Heart Failure Sufficient to Control Heart Rate of Patients?

**Authors:** Juliano Cardoso, Mateus Dressler de Espíndola, Mauricio Cunha, Enock Netto, Cristina Cardoso, Milena Novaes, Carlos Henrique Del Carlo, Euler Brancalhão, Alessandro Lyra Name, Antonio Carlos Pereira Barretto

**Affiliations:** 1 Brazil Hospital Santa Marcelina – Cardiologia, São Paulo, SP – Brazil

**Keywords:** Heart Failure, Heart Rate, Drug Therapy, Medication Adherence, Digoxine, Morbidity & Mortality, Atrial Fibrillation, Pacemaker, Artificial

## Abstract

**Background:**

Studies have shown that heart failure (HF) patients with heart rate (HR) < 70 bpm have had a better clinical outcome and lower morbidity and mortality compared with those with HR > 70 bpm. However, many HF patients maintain an elevated HR.

**Objective:**

To evaluate HR and the prescription of medications known to reduce mortality in HF patients attending an outpatient cardiology clinic.

**Methods:**

We consecutively evaluated patients seen in an outpatient cardiology clinic, aged older than 18 years, with diagnosis of HF and left ventricular ejection fraction (LVEF) < 45%. Patients with sinus rhythm were divided into two groups – HR ≤ 70 bpm (G1) and HR > 70 bpm (G2). The Student’s t-test and the chi-square test were used in the statistical analysis, and a p-value < 0.05 was considered statistically significant. The SPSS software was used for the analyses.

**Results:**

A total of 212 consecutive patients were studied; 41 (19.3%) had atrial fibrillation or had a pacemaker implanted and were excluded from the analysis, yielding 171 patients. Mean age of patients was 63.80 ± 11.77 years, 59.6% were men, and mean LVEF 36.64±7.79%. The most prevalent HF etiology was ischemic (n=102; 59.6%), followed by Chagasic (n=17; 9.9%). One-hundred thirty-one patients (76.6%) were hypertensive and 63 (36.8%) diabetic. Regarding HR, 101 patients had a HR ≤70 bpm (59.1%) and 70 patients (40.93%) had a HR >70 bpm (G2). Mean HR of G1 and G2 was 61.5±5.3 bpm and 81.8±9.5 bpm, respectively (p<0.001). Almost all patients (98.8%) were receiving carvedilol, prescribed at a mean dose of 42.1±18.5 mg/day in G1 and 42.5±21.1mg/day in G2 (p=0.911). Digoxin was used in 5.9% of patients of G1 and 8.5% of G2 (p=0.510). Mean dose of digoxin in G1 and G2 was 0.19±0.1 mg/day and 0.19±0.06 mg/day, respectively (p=0,999). Most patients (87.7%) used angiotensin converting enzyme inhibitors (ACEI) or angiotensin II receptor blockers (ARB), and 56.7% used spironolactone. Mean dose of enalapril was 28.9±12.7 mg/day and mean dose of ARB was 87.8±29.8 mg/day. The doses of ACEI and ARB were adequate in most of patients.

**Conclusion:**

The study revealed that HR of 40.9% of patients with HF was above 70 bpm, despite treatment with high doses of beta blockers. Further measures should be applied for HR control in HF patients who maintain an elevated rate despite adequate treatment with beta blocker. (Arq Bras Cardiol. 2020; 115(6):1063-1069)

## Introduction

Heart failure (HF) is an increasingly frequent syndrome associated with high morbidity and mortality in severe cases, and a common end-stage of heart diseases.^1^

Despite the severity of HF, an effective guideline-based treatment of HF can improve the quality of life and reduce mortality of patients.^2 , 3^However, the prescription of medications known to improve HF patients’ prognosis is still lower than expected, as shown in recent reports.^4 - 6^Among the main causes of the under-prescription of medications to HF patients are hypotension, older age, and fear of potential side effects.^6 , 7^Another possible reason why drug doses that have been proven effective in HF are not achieved is the lack of clear therapeutic objectives, as commonly seen in the treatment of dyslipidemias and hypertension.^8 , 9^Perhaps we should set some clear targets, including a more strict control of heart rate (HR), which has been shown to be an important guide to assess treatment efficacy. The SHIFT study has shown that reducing HR values to less than 70 beats per minute (bmp) can improve the prognosis of HF patients with reduced left ventricular ejection fraction (LVEF) and sinus rhythm. However, HR values in some of our patients are still higher than expected.^10^

In the present study, we aimed to verify whether HF patients with sinus rhythm, attending the outpatient department of a large tertiary hospital in east São Paulo, had controlled HR (i.e., HR≤70 bpm). We also evaluated whether these patients were receiving appropriate drug therapy, in accordance with guidelines on HF management.^2^

## Methods

Consecutive patients with HF and LVEF <45% seen at a cardiology outpatient clinic between January 2016 and March 2017, treated for HF for at least six months participated in the study. We assessed demographic data, etiology of heart disease, heart rhythm, blood pressure, HR, and drug treatment, including the doses achieved of each drug.

Inclusion criteria were age older than 18 years, diagnosis of HF, LVEF <45% and sinus rhythm. Patients were divided into two groups – patients with HR ≤70 bpm (G1) and patients with HR >70 bpm (G2).

Prescription of the three groups of medications proven to change the natural history of HF was evaluated – 1) vasodilators: angiotensin converting enzyme inhibitors (ACEI), 2) angiotensin II receptor blockers (ARBs), 3) spironolactone and beta blockers. Patients with renal dysfunction and persistent hyperkalemia, who did not tolerate 50% of ACEI/BRA, used hydralazine and nitrates.

The dose of ACEI considered was 20mg twice daily or equivalent dose of captopril (150mg per day). The dose of ARBs considered was 100-150 mg per day for losartan, and a full dose of 25mg per day for spironolactone. For beta blockers, the full dose was 25 mg twice a day for carvedilol.^2^We also evaluated the percentage of prescription and dose of commonly prescribed drugs for HF treatment, including digoxin, hydrochlorothiazide, and furosemide.^2^

The study was approved by the Research Ethics Committee of Casa de Saúde Santa Marcelina (approval number 13.10.805).

### Statistical Analysis

For characterization of the study population, continuous variables with normal distribution were described as mean ± standard deviation. Categorical variables were described as number (percentage). The Kolmogorov-Smirnov test was used to verify normality of data distribution (p>0.05 = normal distribution). For group comparisons, continuous variables were described as mean ± standard deviation; and for comparisons of patients’ characteristics, the chi-square or the Fisher exact test was used. All analyses were performed using the Statistical Package for the Social Sciences (SPSS) software.

## Results

A total of 212 consecutive patients were studied; 41 of them were excluded for having atrial fibrillation or a pacemaker implanted. Therefore, 171 patients with sinus rhythm were assessed. Mean age was 63.8 ± 11.8 years, 59.6% were men, mean LVEF was 36.64 ± 7.79% and mean pro-brain natriuretic peptide level was 1663.95 ± 2158.77 pg/mL. An ischemic etiology of HF was identified in 63 patients (36.84%), and 131 patients (76.6%) were hypertensive. Clinical characteristics and HF treatment of patients are described in Table 1 . Doses of prescribed medications are listed in Table 2 .

**Table 1:** – Characteristics and drug treatment of heart failure patients with sinus rhythm (n=171)

Characteristics	
Age (years)	63.80 ± 11.77
**Etiology of heart failure**	
Ischemic	102 (59.65%)
Chagasic	17 (9.9%)
Idiopathic	29 (17%)
**Comorbidities (n)**	
Diabetes mellitus	63 (36.84%)
Hypertension	131 (76.6%)
**Clinical data**	
SBP (mmHg)	119.56 ± 18.69
Heart rate (bpm)	69.81 ± 12.34
NT-proBNP	1663.95 ± 2158.77
**Echocardiographic data**	
LVDD (mm)	61.34 ±7.79
LVSD (mm)	50.33 ± 8.25
LVEF (%)	36.64 ± 6.73
**Drugs prescribed; n (%)**	
Furosemide	90 (52.63%)
Hydrochlorothiazide	25 (14.61%)
ACEI/ARB	150 (87.72%)
Beta blocker	169 (98.83%)
Spironolactone	97 (56.72%)
Hydralazine	30 (17.54%)
Nitrates	42 (24.56%)
Digoxin	12 (7.01%)

*SBP: systolic blood pressure; NT-proBNP: N-terminal pro–B-type natriuretic peptide; LVDD: left ventricular diastolic diameter; LVSD: left ventricular systolic diameter; LVEF: left ventricular ejection fraction; ACEI: angiotensin converting enzyme inhibitors (ACEI), ARBs: angiotensin II receptor blockers.*

**Table 2:** – Mean dose (mg/day) of the drugs prescribed

Medication	Dose (mg/day)
Furosemide	52.31 ± 26.15
Hydrochlorothiazide	26.09 ± 5.10
Enalapril	28.86 ± 12.68
Losartan	87.80 ± 29.80
Carvedilol	42.28 ± 19.65
Spironolactone	25.00 ± 4.77
Hydralazine	96.55 ± 59.35
Isosorbide	53.90 ±15.60
Digoxin	0.19 ± 0.06

*Data expressed as mean ± standard deviation*

Comparisons between G1 and G2 are described in Table 3 .

**Table 3:** – Comparison of clinical data and drug therapies of patients with heart failure and sinus rhythm by heart rate values (≤70bpm vs. >70 bpm)

	G1 (HR ≤70bpm)	G2 HR>70 bpm	p
Patients; n (%)	101 (59.06%)	70 (40.93%)	
Men; n (%)	62 (61.38%)	40 (57.97%)	
**Etiology of heart rate**			
Ischemic	59 (58.41%)	43 (61.64%)	0.938
Chagasic	16 (15.84%)	3 (4.28%)	0.009
Non-ischemic	26 (25.74%)	24 (34.28%)	
**Comorbidities**			
Diabetes mellitus	31 (30.69%)	32 (45.71%)	0.045
Hypertension	82 (81.18%)	50 (71.42%)	0.108
**Clinical data**			
SBP (mmHg)	119.76 ± 17.87	119.29 ± 19.81	0.871
HR (bpm)	61.53 ± 5.26	81.76 ± 9.52	<0.001
NT-proBNP	1625.09 ± 2258.42	1721.80 ± 1999.91	0.822
**Echocardiographic data**			
LVDD (mm)	61.26 ± 7.78	61.46 ± 7.82	0.868
LVSD (mm)	49.84 ± 8.42	51.12 ± 7.92	0.356
LVEF (%)	37.46 ± 6.58	35.46 ± 6.78	0.056
**Doses of the drugs prescribed**			
Furosemide	50.57 ± 25.06	54.74 ± 27.41	0.458
Hydrochlorothiazide	26.92 ± 6.66	25.00 ± 0.00	0.392
ACEI/ARB	29.77 ± 12.38	27.50 ± 12.99	0.361
Betablocker	80.43 ± 33.75	97.22 ± 20.22	0.076
Spironolactone	42.14 ± 18.55	42.48 ± 21.14	0.911
Hydralazine	24.79 ± 4.84	25.35 ± 4.64	0.585
Nitrates	111.11 ± 67.81	72.73 ± 29.11	0.097
Digoxin	55.77 ± 16.21	50.67 ± 13.89	0.325
Furosemide	0.19 ± 0.06	0.19 ± 0.06	0.999

*HR: heart rate; SBP: systolic blood pressure; LVDD: left ventricular diastolic diameter; LVSD: left ventricular systolic diameter; LVEF: left ventricular ejection fraction; NT-proBNP: N-terminal pro–B-type natriuretic peptide.*

## Discussion

Our study showed that 40.93% of patients with HF had a HR above 70 bpm, despite the use of a high dose (>42 mg/day) of carvedilol by more than 98% of patients. Most patients were correctly prescribed with medications that could change the prognosis of the disease. The frequency of prescription of HF drugs was higher than previously described in international registries as well as in the Brazilian registry of heart failure (BREATHE).^4 - 6^Most patients were receiving the target dose of the drugs prescribed as recommended in the guidelines.^2 , 3^

These results allow us to suggest that the main cause of non-prescription of HF drugs by physicians is the lack of attempts to increase the treatment doses. It is important to highlight that the non-prescription of at least 50% of target dose leads to lower protection and higher risk of death and hospitalizations.^6 , 7^

Our data differ from those previously published in registries such as the BIOSTAT-HF, in which only 60% of patients achieved at least 50% of the recommended treatment dose for beta blockers, which has been shown to reduce mortality.^6^Similarly, the Brazilian registry - BREATHE – showed that 83.4% and 63.1% of HF patients were receiving ACEI/ARB and beta blockers, respectively, at hospital discharge.^5^In the QUALIFY registry, although most patients (87.5%) were prescribed ACEI/ARB, only 14.8% achieved the target dose, and 51.8% were using a dose greater than 50% of the target dose for ACEI. In this same study, 27.9% of patients were taking the target dose for beta blockers, and 51.8% were receiving a dose greater than 50% of the target dose for beta blocker.^4^In our study group, 79.09% of patients achieved the recommended dose for ACEI, and 53.63% of them were using enalapril 40 mg/day, and 58.47% achieved the recommended dose for beta blockers (15% of them were receiving a dose greater than 50 mg/day of carvedilol. A large majority (97.27%) of patients were prescribed a dose greater than 50% of ACEI, and 88.88% of patients were receiving a dose greater than 50% of carvedilol ( Figure 1 ).

**Figure 1:** – Percentage of users and mean dose of the prescribed medications for heart failure treatment in 171 patients (mean left ventricular ejection fraction of 36.6%) seen at the cardiology outpatient clinic of Santa Marcelina Hospital, São Paulo, Brazil.

Our study also revealed that many of the patients treated with carvedilol 42.48mg/day, and 40% of the patients with sinus rhythm had a HR greater than 70 bpm ( Figure 2 ). These results are in agreement with the literature, since all studies reporting HR data of HF patients treated with ACEI, beta blockers and spironolactone have shown that a high percentage of them maintain the HR at values above 70 bpm despite treatment. It worth mentioning that in many of these studies, the beta blocker dose used by the patients was lower than 50% of target dose.^6 , 7^

**Figure 2:** – Distribution of heart failure patients with sinus rhythm by heart rate (>70 bpm or <70 bpm).

In the OPTIMIZE-HF registry, that evaluated 10,697 hospitalized patients in the United States, mean HR at hospital discharge was 76 bpm, with no correlation between HR and dose of beta blocker. Patients receiving a dose lower than 25% of the target dose for beta blockers showed a mean HR of 78 bpm, and those who achieved the target dose showed a mean HR of 72 bpm. An elevated HR correlated with the prognosis, with higher morbidity and mortality among patients with HR greater than 70 bpm.^11^At Duke University, most of patients with HF (73%) showed elevated HR (>70 bpm) despite treatment. Elevated HR was associated with higher morbidity and mortality (RR 1.59), and higher treatment cost.^12^Habal et al.^13^reported that the risk of death was 59% higher in patients with a HR above 90 bpm compared with those with HR of 61-70 bpm.^13^The ASCEND-HF study showed that 85% of HF patients had elevated HR (>70 bpm) despite treatment, which was associated with higher mortality.^14^

In our study, although a considerable number of patients showed a HR above 70 bpm, the number was lower than those reported in these previous studies, which may be explained by the different doses of beta blockers used by the patients. In addition, a study on office patients who were receiving carvedilol at a dose near to the target dose reported that 35% of patients with HF had a HR above 70 bpm.^15^

An issue discussed in the literature concerns which is a more important determinant of prognosis, the target dose of beta blocker or the reduction in HR. It is worth pointing out that the HR reduction achieved by beta blockers differs among patients. In the MERIT-HF study, two groups of patients were identified – one group that showed a HR reduction in response to low doses of metoprolol (mean 76 mg/day) and another group that achieve HR reduction in response to high doses of metoprolol (mean 195 mg/day).^16^Such difference may be genetically determined, with some of the patients highly responsive to relatively low doses of medications.^16^The authors pointed out that the reduction in events was not different between the two groups, indicating that HR reduction was a more important determinant to reduction of cardiovascular events compared with the beta blocker dose.^16^Considering HR reduction, the SHIFT study suggested a target HR below 70 bpm,^10^and suggested that HR reduction is also more important than the dose of beta blocker in reducing cardiovascular events.^17^It is of mention that the greater reduction in events is achieved when the HR is reduced to values lower than 64 bpm, as demonstrated in the CHARM and CIBIS-ELD studies.^18 , 19^

A meta-analysis of several clinical trials with beta blockers showed that the prescription of these drugs was associated with a reduction in mortality of 34%, and that the HR reduction was more strongly associated with a reduction in events that the dose of beta blocker.^20^In addition, the study showed that for every reduction in HR of 5 beats/min, the risk of death decreased by 18%, and the dose of beta blocker was not determinant to event reduction, with a reduction in the risk of death of 26% in patients receiving a higher dose, and of 22% in those receiving a lower dose.^20^

The BIOSTAT-HF study and the retrospective analysis of the ACTION-HF data revealed a greater reduction in cardiovascular events in patients treated with a higher dose of beta blocekrs.^6 , 21^In the BIOSTAT-HF study, doses greater than 50% of target dose were associated with greater mortality reduction in 2,516 patients. In the Discussion section, the authors state that they did not detect any difference in the clinical course of patients treated with more than 50% of target dose compared with those treated with the target dose; however, lower doses did not have a protective effect.^6^In the ACTION-HF study, the patients who had a better clinical outcome (greater mortality reduction) were those who achieved HR reduction to lower than 70 bpm with a dose of 50% or more of beta blocker. Patients receiving a lower beta blocker dose showed higher mortality rate. When only patients receiving a low dose of beta blocker were analyzed, those with HR values below 70 bpm showed a better clinical outcome than those with HR values above 70 bpm.^21^

We can conclude that both low doses of beta blocker and a HR above 70 bpm are associated with a worse prognosis. Data of the literature have highlighted the importance of evaluating HR in all HF patients and optimizing the treatment in those with HR above 70 bpm, by either increasing the dose of beta blocker or by prescribing ivabradine in attempt to reduce it, since a HR of more than 70 bpm has been shown an excellent and easy marker of worse course. It is worth remembering that the higher the HF the worse the prognosis. We should be careful to avoid postponing the decision to change the therapy when we see a patient with sinus rhythm and HR above 70 bpm. It is also important to point out that the efficacy of be blockers at low doses has not been proven. Besides, in patients with elevated HR despite treatment with beta blocker, we can use ivabradine, which is a selective *f* current blocker, that reduces HR in patients with sinus rhythm.^2^

One limitation of this study was the fact that it was a single-center study. The strength of this study lies on the fact that it demonstrated that many HF patients treated with adequate dose of beta blocker maintain HR at high levels,^22^which is related to their prognosis.

## Conclusion

Of the patients with sinus rhythm evaluated, 98.83% were prescribed a high dose of beta blocker (42.28 ± 19.65 mg/day), but 40.93% maintained HR at levels above 70 bpm. Further measures should be applied for a better HR control of these patients who maintain an elevated HR despite adequate treatment with beta blocker. In our study group, a high frequency of patients was taking vasodilators, at adequate mean dose.
